# Subgenual cingulate cortical activity predicts the efficacy of electroconvulsive therapy

**DOI:** 10.1038/tp.2016.54

**Published:** 2016-04-26

**Authors:** M Argyelan, T Lencz, S Kaliora, D K Sarpal, N Weissman, P B Kingsley, A K Malhotra, G Petrides

**Affiliations:** 1 Center for Psychiatric Neuroscience, The Feinstein Institute for Medical Research, Manhasset, NY, USA; 2 Division of Psychiatry Research, Zucker Hillside Hospital, Northwell Health, Glen Oaks, NY, USA; 3 Department of Psychiatry, Hofstra Northwell School of Medicine, Hempstead, NY, USA; 4 Department of Radiology, North Shore University Hospital, Northwell Health, Manhasset, NY, USA

## Abstract

Electroconvulsive therapy (ECT) is the most effective treatment for depression, yet its mechanism of action is unknown. Our goal was to investigate the neurobiological underpinnings of ECT response using longitudinally collected resting-state functional magnetic resonance imaging (rs-fMRI) in 16 patients with treatment-resistant depression and 10 healthy controls. Patients received bifrontal ECT 3 times a week under general anesthesia. We acquired rs-fMRI at three time points: at baseline, after the 1st ECT administration and after the course of the ECT treatment; depression was assessed with the Hamilton Depression Rating Scale (HAM-D). The primary measure derived from rs-fMRI was fractional amplitude of low frequency fluctuation (fALFF), which provides an unbiased voxel-wise estimation of brain activity. We also conducted seed-based functional connectivity analysis based on our primary findings. We compared treatment-related changes in HAM-D scores with pre- and post-treatment fALFF and connectivity measures. Subcallosal cingulate cortex (SCC) demonstrated higher BOLD signal fluctuations (fALFF) at baseline in depressed patients, and SCC fALFF decreased over the course of treatment. The baseline level of fALFF of SCC predicted response to ECT. In addition, connectivity of SCC with bilateral hippocampus, bilateral temporal pole, and ventromedial prefrontal cortex was significantly reduced over the course of treatment. These results suggest that the antidepressant effect of ECT may be mediated by downregulation of SCC activity and connectivity. SCC function may serve as an important biomarker of target engagement in the development of novel therapies for depression that is resistant to treatment with standard medications.

## Introduction

Depression is a major public health problem, affecting up to 20% of the US population at least once in their lifetime.^1^ Approximately 60% of the patients do not achieve remission with the initial treatment, and more than one-third of patients will eventually be labeled ‘treatment resistant'.^2, 3^ Electroconvulsive therapy (ECT) is known to be the most effective treatment for treatment-resistant depression (TRD),^4, 5^ yet its mechanism of action remains unknown.^6, 7^

Identification of the neural circuitry associated with the efficacy of ECT may provide data needed to detect the central nervous system targets of effective antidepressant treatment, and therefore be important for the development of new interventions with similar efficacy but reduced side effects.^8^ To date, however, neuroimaging studies have failed to unambiguously identify functional changes in the brain that correspond to the therapeutic effects of ECT.^9^ Several studies utilizing positron emission tomography (PET) to measure regional glucose metabolism have suggested that ECT reduces prefrontal activity,^10, 11, 12, 13, 14, 15, 16^ but these results have not been consistently replicated.^17, 18, 19^ The interpretation of these studies is limited by the relatively poor temporal resolution of this technique, as well as (in some instances) the relatively gross spatial resolution of the available scanners. Studies of regional cerebral blood flow, using PET or single-photon emission computed tomography (SPECT), have been even more variable (reviewed by Abbott *et al.*),^20^ with no consistently replicated treatment targets identified.

Resting-state functional magnetic resonance imaging (rs-fMRI) provides a new opportunity to assay both regional function and long-range connectivity, and possesses improved temporal and spatial resolution relative to PET and SPECT. To date, no study has assessed fractional amplitude of low frequency fluctuations (fALFF) measures in the context of a longitudinal ECT study. fALFF quantifies local dynamic fluctuations in the BOLD signal by conducting Fourier transformation and measuring power in low frequency ranges (typically 0.01–0.08 Hz). It is called fractional, since low frequency power is divided by the overall power calculated across all frequency ranges. fALFF has several advantages for the study of rs-fMRI in ECT. First, fALFF permits an unbiased examination of functional activity across the whole brain. Second, it effectively suppresses non-specific signal components, such as physiological noise.^21^ Third, fALFF has been shown to correlate with regional glucose metabolism as measured by PET,^22, 23^ and therefore can be used to test the hypothesis that ECT reduced prefrontal activity, as suggested by the prior PET studies cited above.

**Figure 1:**
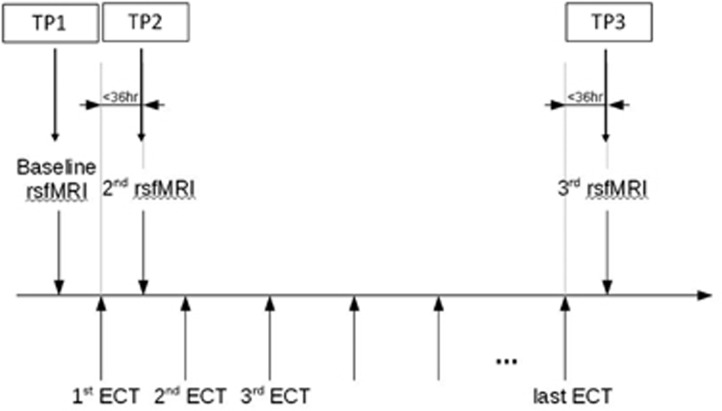
Study design. Patients underwent a course of bifrontal ECT (3 times per week) and were scanned 3 time points during treatment. They were scanned at baseline, within 36 h after the first ECT treatment and within 36 h after the last treatment. The last ECT treatment was obtained at remission or achieved after the 8th ECT treatment if the patient had not remitted (ECT course might have continued for clinical reasons). ECT, electroconvulsive therapy; TP, time point.

Therefore, we conducted a longitudinal rs-fMRI study measuring fALFF in patients initiating a trial of ECT for a major depressive episode, with the primary aim to detect changes in regional neural activity reflecting ECT-induced improvements in mood. Sixteen patients and 10 healthy comparison subjects underwent rs-fMRI at three time points (TP): before the first ECT session (TP1); within 36 h after the first ECT (TP2); and within 36 h after the last or 8th ECT, whichever occurred first (TP3) (Figure 1). To detect treatment-associated networks more broadly, we utilized the top result from the fALFF analysis to define a seed for functional connectivity analysis. Since some prior studies have been confounded by heterogeneity with respect to timing of scans, we rigorously conducted scanning immediately before the first ECT treatment and within a 36-h window after the final ECT treatment. Unlike prior studies, we also included an intermediate scanning session after the first ECT treatment to account for acute effects of seizure and anesthesia on scanning parameters. We also examined healthy comparison subjects, to control for effects of time, as well as to determine if ECT-induced changes represented a ‘normalization' of brain function in remitted patients.

## Materials and methods

### Participants and study design

Sixteen depressed patients (48.5±13.6 years; six females) underwent rs-fMRI at three TPs: before the first ECT session (TP1); within 36 h after the first ECT (TP2); and within 36 h after the last or eighth ECT, whichever occurred first (TP3) (Figure 1). Inclusion criteria for patients were: (1) between 18 and 70 years of age; (2) DSM-IV diagnosis of Major Depression, unipolar without psychotic features, or Bipolar I or Bipolar II Depression without psychotic features confirmed by SCID-IV interview; (3) pretreatment 24-item Hamilton Rating Scale for Depression (HAM-D) score⩾21; (4) pretreatment score of at least 20 on the MADRS; (5) ECT was clinically indicated (at least 2 failed adequate antidepressant trials in the past); (6) competency to provide informed consent. Exclusion criteria were: (1) current diagnosis of delirium, dementia or amnestic disorder; (2) diagnosis of mental retardation; (3) baseline Mini Mental State Exam (MMSE) score<21, or a total score falling 2 s.d. below the age- and education-adjusted mean (whichever is less); (4) any active general medical condition or central nervous system disease, which can affect cognition or response to treatment; (5) current (within the past 3 months) diagnosis of active substance dependence, or active substance abuse within the past week; (6) ECT within the prior 3 months.

During the course of the study, patients' symptoms were assessed with the HAM-D before every ECT; remission was defined as two consecutive HAM-D ratings⩽10. Patients received on average 6.4 ECT before their final MRI session (range: 4–8). Ten patients reached remission before the eighth ECT (or 2.5 weeks, given three ECT per week) and they were scanned for the third time after the last ECT session, when they fulfilled remission criteria. Fourteen of the patients had >50% reduction in HAM-D at TP3. ECT was performed with bifrontal placement on a Thymatron System IV (Somatics, Lake Bluff, IL, USA) under general anesthesia. Ten out of sixteen patients received methohexital 1 mg kg^−1^ while six patients received ketamine 1 mg kg^−1^ as an inducing agent. We conducted *post hoc* analyses, indicating no significant effect of anesthesia type on our results (see Supplementary Material). All patients received succynilcholin 1 mg kg^−1^ as a muscle relaxant. Patients received ECT 3 times a week using 150% of seizure threshold energy. All patients started ECT treatment as inpatients and their psychotropic medications were discontinued. The only psychotropic medication allowed during the study was lorazepam up to 3 mg per day for anxiety or insomnia.

Ten healthy age-matched volunteers (45.6±13.1 years; five females) were recruited to serve as a control population for the imaging measures. The controls underwent the same imaging protocol with similar TPs as the patients.

Written informed consent was obtained from all participants in accordance with the guidance of the North Shore—LIJ Health System Institutional Review Board, which approved this study.

Demographic details of patients and controls are listed in Supplementary Table S1 in the Supplement.

### MRI protocol

MR imaging exams were conducted at North Shore University Hospital on a 3T GE HDx scanner (General Electric, Milwaukee, WI, USA). For image registration, we acquired anatomical scans in the coronal plane using a three-dimensional spoiled gradient sequence (TR=7.5 ms, TE=3 ms, matrix=256 × 256, FOV=240 mm), producing 216 contiguous images (slice thickness=1 mm) through the whole head. We acquired rs-fMRI scans comprising a total of 150 echo-planar imaging volumes with the following parameters: TR=2000 ms, TE=30 ms, matrix=64 × 64, FOV=240 mm, slice thickness=3 mm, 40 continuous axial oblique slices (one voxel=3.75 × 3.75 × 3 mm). During the acquisition, all subjects were instructed to ‘close their eyes and not think of anything in particular.' We acquired 5-min rs-fMRI runs four times at each TPs to increase our statistical power (altogether 20 min of rs-fMRI at each time point). At TP 1 and TP2, we scanned all 16 individuals, only 13 patients were available for scanning at TP3.

### Image processing

We used script libraries derived from the 1000 Functional Connectomes Project (http://www.nitrc.org/projects/fcon_1000;^24^ these libraries, based in FSL (http://www.fmrib.ox.ac.uk) and AFNI (http://afni.nimh.nih.gov/afni), were used for preprocessing along with a lab-developed script in the R statistical language (Vienna, Austria) for additional analysis as described below. Standard preprocessing and motion analysis steps are detailed in the Supplement.

### Image analysis

Image analysis was carried out in two steps: voxel-wise fALFF and seed-based connectivity analysis. In the analysis, we calculated these measures independently in the four 5-min rs-fMRI runs (no concatenation) and used them as intra-subject repetitions in the following higher order statistical tests. First, fALFF images were calculated in each run. In this process, utilizing Fourier Transformation of the unfiltered signal in every voxel, we calculated the power of BOLD signal in the low frequency range of 0.01–0.08 Hz and divided it by the power of BOLD signal across the entire frequency range (0–0.25 Hz); this approach has proved to be an effective way to correct for non-physiological noise in the BOLD signal.^21^ *Z*-normalized fALFF maps were assessed with voxel-wise statistical parametric mapping (SPM5, London, UK; family-wise error-corrected *P<*0.05); for our primary analysis, we compared baseline (TP1) with the third time point (TP3). We implemented repeated measures of ANOVA with a flexible factorial model, with three factors: subjects (independent across levels), TPs and runs (dependent across levels), and tested TP1>TP3 and TP1<TP3 with appropriate contrast over the main effect time. We applied a conservative threshold for statistical significance, reporting clusters where at least 10 voxels survived family-wise error correction at *P<*0.05 level. As a follow-up exploratory analysis, to more broadly examine treatment-relevant networks, we also report clusters meeting an uncorrected *P<*0.001.

Next, we conducted seed-based correlation analysis derived from the most significant voxels identified from the fALFF analysis. Specifically, we used 3 × 3 × 3 voxel size box around the peak coordinates of the findings of the voxel-based analysis (fALFF). Individual seed-based correlation maps were created and Fisher transformed, followed by SPM5 voxel-based analysis, utilizing the strict statistical threshold of false discovery rate corrected *P<*0.05 with minimum 10 voxels.

In follow-up *post hoc* analyses, we repeated both fALFF and seed-based connectivity analyses including all the three TPs from both patients and healthy controls (HC). To simplify interpretation and display of data, the four runs were averaged during these analyses in the result.

## Results

Patients' mood improved significantly with ECT treatment from TP1 to TP3 (HAM-D decrease: 17.9±5.1, *t*=9.1, degree of freedom (df)=30, *P<*0.001, HAM-D % change: 63.9±16.6% mean HAM-D at baseline: 28.2, at TP2: 21.6, at TP3: 10.3).

**Figure 2:**
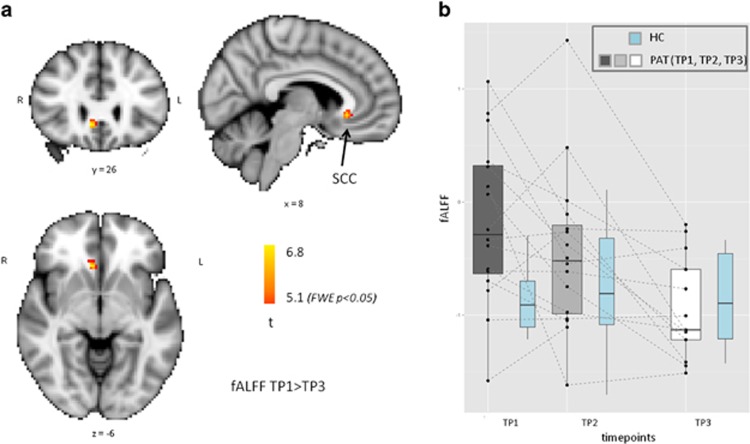
Decreases in fALFF between TP1 to TP3. (**a**) Voxel-based statistics across whole brain. (**b**) *Post hoc* analysis of the primary result including all the three time points from both patients and healthy controls (with blue). These results show that fALFF was higher at baseline in depressed patients, and it was normalized during the course of ECT (see statistics in the text). ECT, electroconvulsive therapy; fALFF, fractional amplitude of low frequency fluctuation; TP, time point.

**Figure 3:**
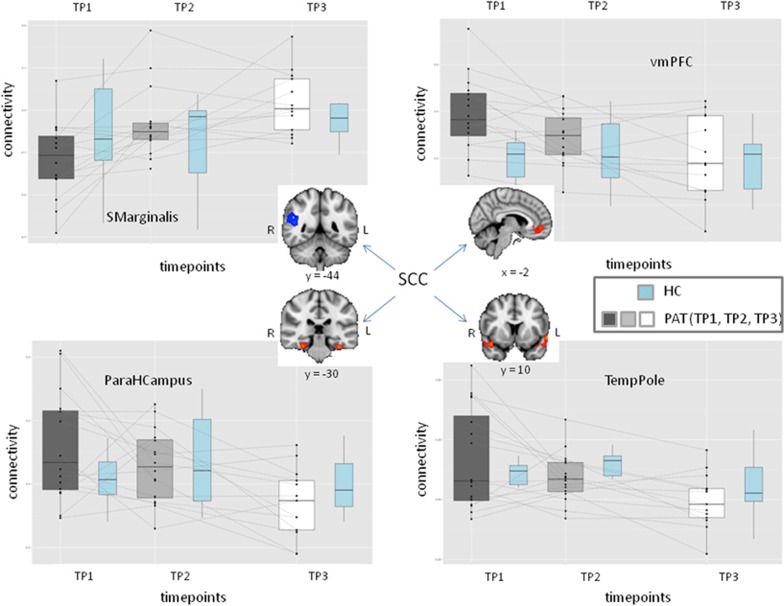
Functional connectivity of subcallosal cingulate cortex (SCC, the most significant region of the fALFF analysis) was used as a seed region to evaluate functional connectivity. Between TP1 and TP3, there were significant decreases in the correlation in three regions: ventromedial prefrontal cortex (vmPFC), bilateral parahippocampal gyrus (HCampus) and bilateral temporal pole (TempPole). We also detected increased correlations in the right supramarginal gyrus (Table 1b). Blue box plots represent normal values measured on the respective areas and time points. ECT, electroconvulsive therapy; fALFF, fractional amplitude of low frequency fluctuation; TP, time point.

Whole brain voxel-wise analysis revealed a significant change in fALFF from pre- to post-treatment TPs in the subcallosal cingulate cortex (SCC) (Figure 2a). *Post hoc* analysis of SCC across both groups (patients and controls) and all three TPs revealed that fALFF was significantly higher at baseline in depressed patients compared with HC (*t*=3.43, df: 21.4, *P*=0.002; Figure 3a). Note that the HC value (blue color in Figure 2b) did not change across the 3 TPs (F_1,25_=0.002, *P*=0.97). During the course of ECT, patients' fALFF not only decreased, but compared with control data, it was normalized; at TP3 the difference between HC and major depressive disorder patients was non-significant (*t*=038, df: 15,4, *P*=0.7). The elevated baseline fALFF in SCC significantly correlated with response (*r*=0.52, *t*=2.25, df: 14, *P*=0.04), the higher baseline value predicted better response. The fALFF decrease from TP1 to TP3 had a trend level correlation with clinical improvement (*r*=0.51, *P*=0.08, *t*=1.94, df=11).

Using the exploratory threshold of the primary TP3 versus TP1 analysis, fALFF of other key fronto-limbic regions implicated previously in depression^25^ (bilateral insula, anterior cingulate, dorsolateral prefrontal cortex (PFC) and hippocampus) were also decreased (Table 1a, Supplementary Figure S1). No regions were found which increased fALFF during the course of ECT treatment, even at this lower threshold. *Post hoc* analyses of these additional regions across all TPs and subjects are in the Supplementary Figure S2 in the Supplement. Most of these regions demonstrated a pattern of change over time that was similar to the SCC, with the majority of the reduction observed between TP2 and TP3. However, it is notable that the hippocampus demonstrated all of the reduction (on average) between TP1 and TP2, indicating that fALFF changes in that structure may be a result of seizure and/or anesthesia. In addition, it should be noted that the dorsolateral prefrontal cortex fALFF values began in the normal range and then were driven far below that range after ECT.

We used the SCC cluster (the most significant region of the fALFF analysis) as a seed region and generated individual correlation maps (Supplementary Figure S3 in the Supplement). The comparison of these maps between TP1 and TP3 revealed significant decrease in the correlation between SCC and three regions—ventromedial PFC (vmPFC), bilateral parahippocampal gyrus and bilateral temporal pole. We also detected increased correlations in the right supramarginal gyrus (Table 1b, Figure 3). The *post hoc* analysis revealed that in regions where we measured decreased correlations between TP1 and TP3, the correlation values were originally positive (*P<*0.001, one sample *t*-test on Fisher transformed values) and in the case of vmPFC and hippocampus values were higher than normal (*P<*0.001, *t*=−3.8, df=23.5 and *P*=0.048, *t*=−2.08, df=22.9, respectively). These numbers were trending down to zero in the hippocampus and in the temporal pole (Figure 3). In the case of vmPFC it was normalized (higher than normal at baseline (*r*~0.4) which trended to normal values (*r*~0.2) (Figure 3). By contrast, *post hoc* analysis in the supramarginal cortex revealed that correlations were significantly negative at baseline ((*P<*0.001, one sample *t*-test) and increase in these values meant decreasing correlation in absolute value. Therefore all four of these regions had weaker relationships with SCC at the end of treatment. Notably, none of the connectivity changes in this analysis correlated with clinical response and none of the baseline connectivity values predicted response.

## Discussion

We found that the SCC demonstrated higher BOLD signal fluctuations at baseline in depressed patients and fALFF decreased over the course of treatment (Figure 2). These data are consistent with previous findings implicating the central role of the SCC in the pathophysiology of depression and its treatment. Previous studies of the pharmacologic and psychotherapeutic treatment of depression have provided converging evidence that depressed patients display higher SCC activity at baseline, and that reduction in SCC activity corresponds with clinical improvement.^26, 27, 28, 29, 30, 31, 32, 33^ Similarly, catecholamine depletion causes increased activity in the SCC, in correlation with increasing severity of depressive symptoms.^34^ Notably, these prior studies utilized PET or SPECT technologies. To our knowledge, the present study is the first report demonstrating regionally specific SCC effects of treatment with ECT, and is also the first to demonstrate that fALFF analysis of rs-fMRI data can yield these results.

Furthermore, higher baseline SCC fALFF values predicted better clinical response to ECT treatment, which if replicated could lead to an MRI-based prognostic biomarker. Interestingly, SCC hyper-activation has consistently predicted poor response with other treatment modalities and was consistently reported as elevated in TRD.^29, 35^ This inverse correlation between SCC and response to conventional treatments implies that most forms of treatment cannot consistently modify the activity in this area. Speculatively, it is possible that any intervention which reliably decreases SCC activity would have clinical efficacy in TRD. Indeed, direct de-activation of the SCC is not only possible through deep brain stimulation (DBS), but was shown effective in a subset of severely treatment-resistant population.^29, 36^ Similarly, our results suggest that ECT is able to decrease SCC activity as well. This is an excellent example how target identification of a mechanism of action or a neuronal node in a neuropsychiatry disorder can help to develop novel therapies.^8^ We are aware of the negative outcomes of the recently conducted SCC-targeted DBS clinical studies. Based on this study it can be speculated that a clinical DBS or ECT study on preselected patients with high baseline SCC activity would be more likely to yield positive results. In addition, ECT may be more effective for decreasing SCC activity due to its more global stimulation compared with the current spatial limitations of DBS targeting.

It is notable that hippocampus was the most sensitive to seizure as indicated by its dramatic reduction in fALFF from TP1 to TP2 (Supplementary Figure S2). This observation is consistent with the notion that the hippocampus is one of the most seizure sensitive parts of the human brain, giving it a central role in seizure therapies. An alternative explanation is also possible; since the HAM-D values decreased significantly between TP1 and TP2 (28.2–21.6), the hippocampal changes may reflect mood-related improvements. Indeed in a recent study hippocampal volume was shown to correlate with overall ECT-related clinical response.^37^

The strong modulation of fALFF values in the SCC is not necessarily the direct effect of ECT, but could be an indirect effect mediated through other regions of the emotion regulation network. To explore this question, we conducted a seed-based analysis to measure functional correlations from the SCC. vmPFC, bilateral parahippocampal gyrus and bilateral temporal pole decreased their connectivity with SCC while right supramarginal gyrus connectivity increased significantly between TP1 and TP3 (Table 1b, Figure 3). It is intriguing that vmPFC, bilateral parahippocampal gyrus and bilateral temporal lobe have strong bidirectional neuroanatomic connectivity with SCC.^38^ *Post hoc* analysis revealed that all of these changes in connectivity were in the direction of loss of relationship, meaning that correlation was getting closer to zero after ECT. These results fit very well with previous observations found in depression and ECT treatment; previous findings have indicated that depression is a ‘hyper-connectivity' syndrome, with increased connectivity among key regions implicated in mood regulation.^39^ In addition, recent rs-fMRI studies with ECT treatment^40, 41, 42^ found that connectivity decreased,^40^ or the anti-correlation diminished^41^ between key midline frontal regions such as ACC and SCC and other key cognitive control areas like dorsolateral PFC. Leaver *et al.*^43^ also reported normalization of dysconnectivity between ventral striatum and ventral default mode network. In our results two of these connectivities, bilateral parahippocampal gyrus and vmPFC, were hyperconnectivities at baseline and connectivity normalized on treatment. These regions are implicated in emotional regulation and appraisal^44, 45^ and thought to be primarily responsible for automatic regulation of emotion, in contrast to regions more dorsal and lateral in frontal regions, which are involved in voluntary regulation.^46^ Furthermore these regions are part of the default mode network. Hyperactivity of this network is thought to intensify self referential thoughts and make patients unable to terminate rumination, thereby prohibiting availability of resources for voluntary control of emotions. The third region, bilateral temporal pole, is less frequently discussed in the literature, but an increasing amount of evidence indicates its key role in emotional and social processing (for review see the study by Olson *et al.*^47^).

The exact meaning of fALFF and its relation to the corresponding connectivity measures is not fully understood. In principal, these measures should be independent. In a recent study, however, healthy controls showed strong region-specific correlation between ALFF and voxel-based connectivity strength in the cingulate cortex, superior temporal cortex, insula, medial frontal cortex, parahipppocampal cortex and basal ganglia.^48^ This means that our results suggest either that the altered regional neurophysiology results in lower ALFF and altered connectivity, or that altered incoming connectivities influence the local low frequency fluctuations. The present study is limited in its ability to disentangle these two mechanisms.

We acknowledge several additional limitations in this study. First, the lack of correlation between clinical measures and changes in connectivity can be due to the low sample size. Second, the use of ketamine as an anesthetic agent in six of our cases is a potential confound. Importantly, we reanalyzed our data in the methohexital-only subpopulation, and observed the same results (Supplementary Figure S4 in the Supplementary Materials), suggesting that our findings are not driven by use of ketamine as an anesthetic agent in a subset of patients. In addition, while a strength of our study was the uniformity of electrode placement (bifrontal), our ability to generalize the results to other types of ECT electrode placements is limited. Further studies with the more common unilateral placement are required.

In summary, the present study provides evidence that SCC and its correlated regions (vmPFC, parahippocampal gyrus and temporal lobe) are central in the mechanism of action of the bifrontal ECT. We showed here that fALFF could be used as a sensitive marker of SCC activity, which not only correlates with mood states, but also predicts response to ECT. In contrast to other treatment modalities, hyper-activated SCC seems to be a potential therapeutic target for ECT treatment.

## Figures and Tables

**Table 1:** fALFF and seed-based imaging analysis results

*Regions*	*Peak MNI coordinates*	*Peak* t*-value*	*Cluster size (*t*>5.1) FWE*, P<*0.05*	*Cluster size (*t*>3.1) uncorr.*, P<*0.001*
*(a) fALFF decrease during the course of ECT*				
Subcallosal cingulate cortex	8, 26, −6	6.81	32	271
Right cerebellum	42, −62, −32	5.84	9	173
Right hippocampus	26, −6, −30	5.46	1	145
Left DLPFC	−34, 34, 30	5.37	3	136
Right Insula	46, 0, 2	5.28	1	129
Left Insula	−38, −16, −6	5.13	1	610
Anterior cingulate cortex	6,8,22	5.12	1	187

*Regions*	*Peak MNI coordinates*	*Peak* t*-value*	*Cluster size (voxels)* P<*0.05 FDR and cluster size>100*	*Direction of change*
*(b) Regions with altered SCC correlations*				
Ventromedial prefrontal cortex	−2, 52, −10	4.93	415	↓
Left parahippocampal gyrus	−34, −32, −26	5.03	428	↓
Right parahippocampal gyrus	30, −28, −24	5.28	168	↓
Left temporal pole	−60, 8, −6	5.82	387	↓
Right temporal pole	44,10,−18	4.93	563	↓
Right supramarginal cortex	46, −44, 14	4.85	129	↑

Abbreviations: ECT, electroconvulsive therapy; DLPFC, dorsolateral prefrontal cortex; fALFF, fractional amplitude of low frequency fluctuation; FDR, false discovery rate; FWE, family-wise error; MNI, Montreal Neurological Institute; SCC, subcallosal cingulate cortex.
