# Supplementary material for: Subgenual cingulate cortical activity predicts the efficacy of electroconvulsive therapy
Source: Transl Psychiatry. 2016 Apr 26;6(4):e789–. doi: 10.1038/tp.2016.54 (PMC4872412; doi:10.1038/tp.2016.54)
Supplement: Supplementary Information [file tp201654x1.docx]

Supplementary Material

**Methods**

*Movement analysis*

**“**Scrubbing” individual scans evidencing significant motion is not applicable for fALFF, which requires consecutive scans for analysis in the frequency domain. Therefore, we investigated the potential effects of motion on functional connectivity by examining relative motion displacement during the rs-fMRI exam. To express instantaneous head motion as a scalar quantity, we used the empirical formula detailed in Power et al. ^1^ FD_i_ =∣Δd_ix_∣ +∣Δd_iy_∣ +∣Δd_iz_∣ +∣Δα_i_∣+∣Δβ_i_∣ +∣Δ_γi_∣, where Δd_ix_ =d_(i−1)x_−d_ix_, and similarly for the other rigid body parameters [d_ix_ d_iy_ d_iz_ α_i_ β_i_ γ_i_]. Rotational displacements were converted from degrees to millimeters by calculating displacement on the surface of a sphere of radius 50 mm, which is approximately the mean distance from the cerebral cortex to the center of the head.

*Image preprocessing*

Standard preprocessing included removal of the first four “dummy” scans, motion correction, and spatial smoothing (6-mm FWHM Gaussian kernel). Each individual's 4D time series data were regressed on eight predictors: white matter (WM), cerebrospinal fluid (CSF), and six motion parameters. Consistent with our prior work ^2^ and others ^3^, we did not regress out global signal because it would have interfered with the connectivity strength calculation. Standard registration and normalization to MNI152 space (2 mm) was employed, with the resulting transformation applied to each individual's functional dataset (12-parameter affine transformation). For seed based correlation analysis*,* the time series were then high- and low-pass filtered (cutoff frequencies were 0.005 Hz and 0.1 Hz, respectively) which was followed by linear detrending. This filtering step was not applicable to fALFF which is calculated as a ratio of the power of the BOLD signal across low and high frequency domains ^4^. The seed for functional connectivity analysis was derived from the most significant cluster of the voxel-wise fALFF analysis.

**Results**

*Movement analysis*

In healthy controls (HC), average frame-wise displacements (FD) were 0.16 ± 0.08, 0.14 ± 0.06 and 0.13 ± 0.06 mm at TP1, TP2 and TP 3, respectively In patients, FD values at TP1, TP2, and TP3 were 0.22 ± 0.14, 0.23 ± 0.16 and 0.28 ± 0.14 mm, respectively. Results of a repeated measures ANOVA of frame-wise displacement values with factors time (dependent) and group (independent), showed that patients had significantly higher FD values than controls (F_1,61_=9.9, p=0.002). Subsequent analyses showed that time had no main effect (F_1,61_=0.36, p=0.55) and there was also no interaction between factors (F_1,61_=1.0 p=0.32). We observed no significant correlations between movement and baseline subcallosal cingulate cortex (SCC) values (t=0.29, df=13, p=0.78, r=0.08) or between movement parameters and SCC values across all time points (t=0.12, df=38, p=0.90, r=0.02). Overall, our movement analysis indicates that while depressed patients showed increased movement during scans, it is unlikely that movement drives our results. In our longitudinal analysis patients and healthy controls did not show differences in movement over time. Most importantly, there were no correlations between fALFF findings and movement parameters.

*ECT related fALFF changes: post-hoc analysis (Table 1 A)*

In addition to our finding that fALFF decreased significantly the SCC between TP1 and TP3, we observed six other areas with similar decreases in fALFF (p<0.05, FWE corrected, Figs. S1 and S2, Table 1 A). These regions were categorized based on their baseline fALFF values (indicated with blue on Figure S2). The anterior cingulate cortex and right hippocampus, like the SCC, had higher than normal fALFF at baseline (t=2.93, df=21.65, p=0.008 and t=2.47, df=19.88, p=0.02 respectively), which normalized during the course of ECT treatment (at TP3 there were no significant differences). Unlike the SCC, however, baseline fALFF of the ACC and right hippocampus did not correlate with clinical response. The dorsolateral prefrontal cortex (DLPFC), which showed no baseline difference in fALFF (p=0.86), “denormalized” during the course of ECT (Figure S2). After one ECT treatment, patients had lower fALFF in DLPFC than HC (p=0.087); at the end of the ECT course this difference significantly increased (t=-3.09, df=16.61, p=0.007). The remaining three areas, right and left insula and right cerebellum, shown in Figure S2, were normal at baseline, showed significant reductions in fALFF during the ECT course, but they remained in normal range as a group.

The effect of anesthetic agent

Subjects were treated with two different anesthetic agents in our study: methohexital or ketamine. Ketamine has antidepressant properties as well ^5,6^ and has been implicated in causing connectivity changes in resting state fMRI studies ^7–9^. Therefore we re-examined the main imaging analysis (fALFF between TP1 and TP3) in the subgroup of patients with methohexital anesthesia (n=10). The main findings remained significant at the same threshold (see Fig S4A, in comparison to Figure S1A). We also conducted post-hoc analyses on the SCC by dividing the patients according to their anesthetic agents. Repeated measures ANOVA of the SCC showed that there was no interaction effect between time (TP1, TP2 and TP3) and anesthesia agents (F_1,39_=1.36, p=0.25) (Fig S4 B). Our post-hoc analyses also showed that if we excluded the 6 patients with ketamine anesthesia from our analysis, our results remained strongly significant (t=3.78, df=17.2, p=0.001) (Fig S4 B). The fALFF change in the methohexital-only subgroup correlated with the change in the HAM-D at a similar level as the whole sample (r=0.47, n=10, p=0.17 versus r=0.51, p=0.08, n=13 (the three missing value at TP3 are all patients with ketamine anesthesia)). We acknowledge that due to the limited size of subgroups these analyses are not powered enough to rule out interaction, but it serves to demonstrate that it is not ketamine that drives our effects.

**References**

1 Power JD, Cohen AL, Nelson SM, Wig GS, Barnes KA, Church JA *et al.* Functional Network Organization of the Human Brain. *Neuron* 2011; **72**: 665–678.

2 Argyelan M, Ikuta T, DeRosse P, Braga RJ, Burdick KE, John M *et al.* Resting-state FMRI connectivity impairment in schizophrenia and bipolar disorder. *Schizophr Bull* 2014; **40**: 100–110.

3 Yang GJ, Murray JD, Repovs G, Cole MW, Savic A, Glasser MF *et al.* Altered global brain signal in schizophrenia. *Proc Natl Acad Sci USA* 2014; **111**: 7438–7443.

4 Zou Q-H, Zhu C-Z, Yang Y, Zuo X-N, Long X-Y, Cao Q-J *et al.* An improved approach to detection of amplitude of low-frequency fluctuation (ALFF) for resting-state fMRI: Fractional ALFF. *Journal of Neuroscience Methods* 2008; **172**: 137–141.

5 aan het Rot M, Zarate Jr. CA, Charney DS, Mathew SJ. Ketamine for Depression: Where Do We Go from Here? *Biological Psychiatry* 2012; **72**: 537–547.

6 Berman RM, Cappiello A, Anand A, Oren DA, Heninger GR, Charney DS *et al.* Antidepressant effects of ketamine in depressed patients. *Biological Psychiatry* 2000; **47**: 351–354.

7 Gass N, Schwarz AJ, Sartorius A, Schenker E, Risterucci C, Spedding M *et al.* Sub-Anesthetic Ketamine Modulates Intrinsic BOLD Connectivity Within the Hippocampal-Prefrontal Circuit in the Rat. *Neuropsychopharmacology* 2014; **39**: 895–906.

8 Liao Y, Tang J, Fornito A, Liu T, Chen X, Chen H *et al.* Alterations in regional homogeneity of resting-state brain activity in ketamine addicts. *Neuroscience Letters* 2012; **522**: 36–40.

9 Scheidegger M, Walter M, Lehmann M, Metzger C, Grimm S, Boeker H *et al.* Ketamine Decreases Resting State Functional Network Connectivity in Healthy Subjects: Implications for Antidepressant Drug Action. *PLoS ONE* 2012; **7**: e44799.

Table S1. Baseline characteristics of the participants

| **Characteristics** | **MDD** | **HC** |
| --- | --- | --- |
| N | 16 | 10 |
| Age (y) | 48.5 ± 13.6 | 45.6 ± 13.1 *(ns)* |
| Sex: M/F | 11/5 | 5/5 *(ns)* |
| Anesthetic agent: K/M | 6/10 |  |
| Baseline HRSD | 28.2 ± 5.6 |  |
| HRSD_((T1_-_T3)/T1)_ (%) | 63.9 ± 16.6 |  |
| Diagnosis: BP/MDD | 3/13 |  |
| Previous ECT/no past ECT | 3/13 |  |
| Treatment # at T3 (range) | 6.4 ± 1.5 (4-8) |  |

*MDD: major depressive disorder, HC: healthy control, ns: no significant difference between MDD and HC.* *K: ketamine, M: methohexital, T1: first time point (baseline), T3: third time point (after the course of ECT treatment but no later than 8 treatments. Treatments were 3 times/week Monday, Wednesday and Friday.BP: bipolar disorder (type I), MDD: major depressive disorder (all patients were treated for depression)*

Figure S1.


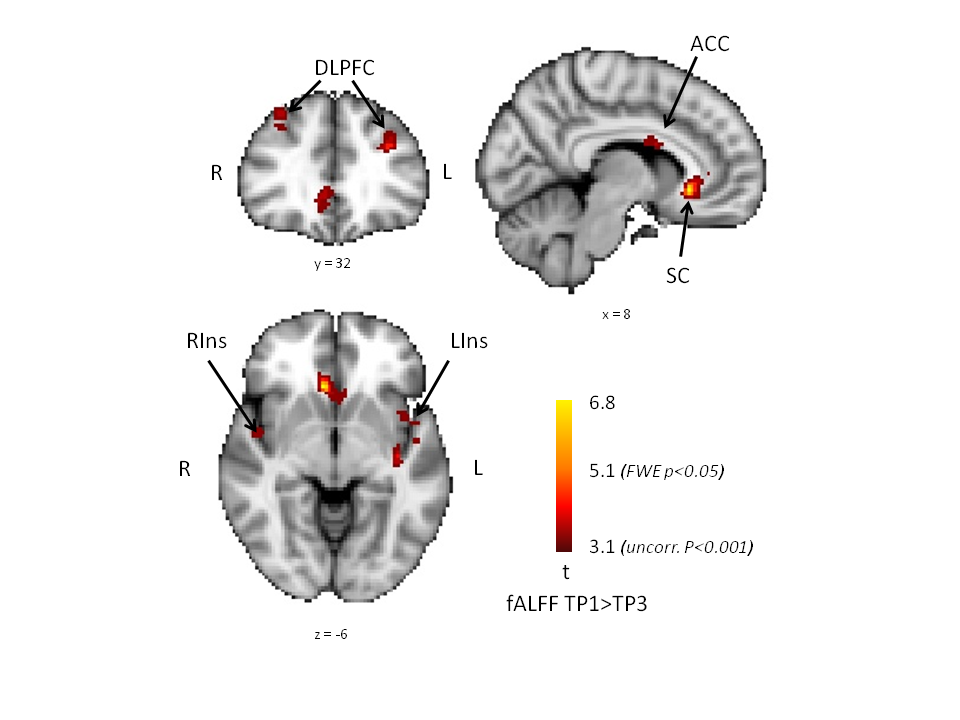


Regions that showed differences in fALFF between TP3 and TP1 are displayed at an exploratory threshold (p<0.001, uncorrected). SCC: subcallosal cingulate cortex; RIns: right insula; LIns: left insula; ACC: anterior cingulate cortex (ACC); DLPFC: dorsolateral prefrontal cortex

Figure S2.


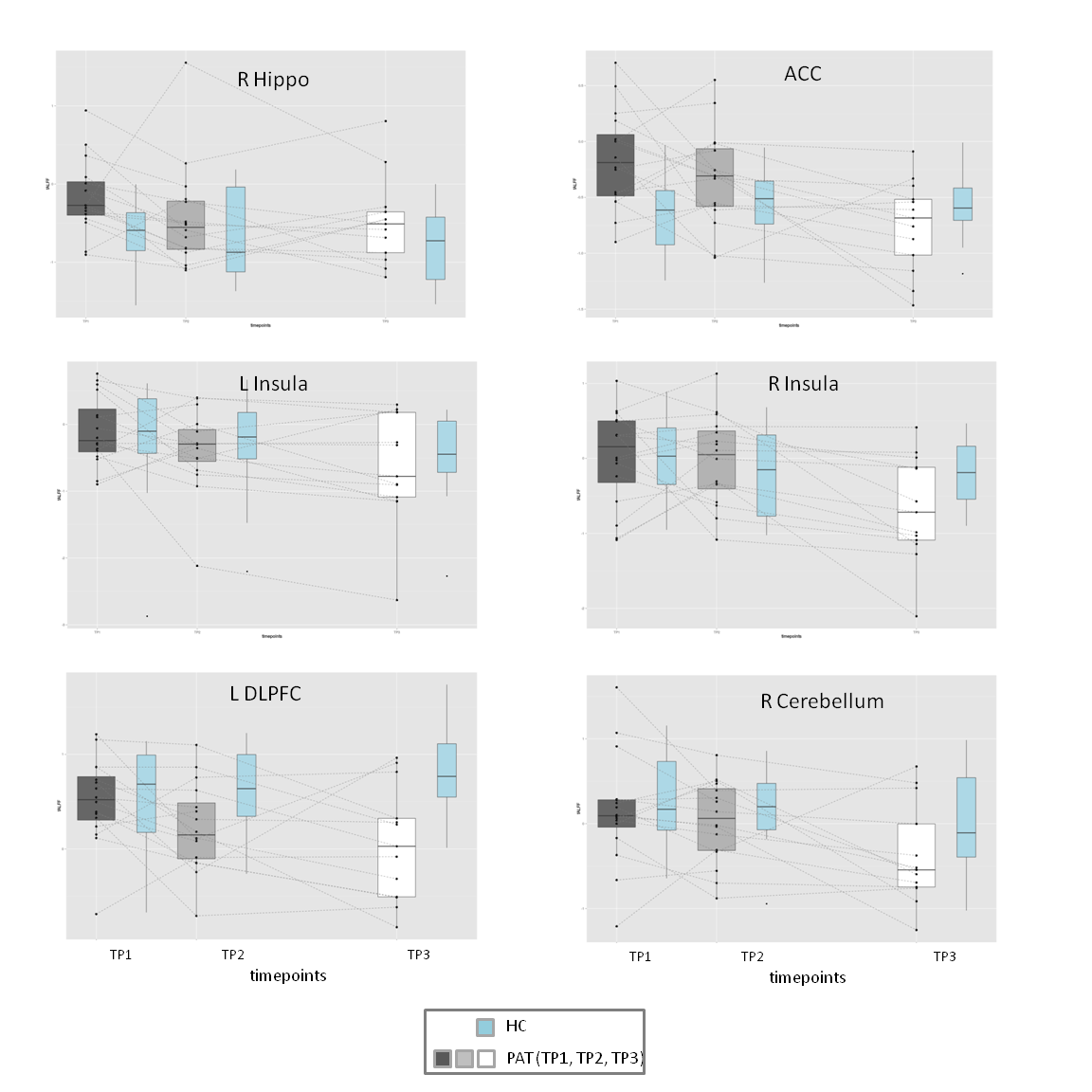
Post hoc analysis of the exploratory findings from Table 1 A and Figure S1. Dark gray is TP1, gray is TP2 and white is TP3 in patients. Blue shadow indicates confidence interval (95%) of the corresponding values of ten healthy controls at TP1, TP2 and TP3 respectively. Normal values help to interpret results: hippocampus and vMPFC were normalized while DLPFC was de-normalized.

Figure S3.


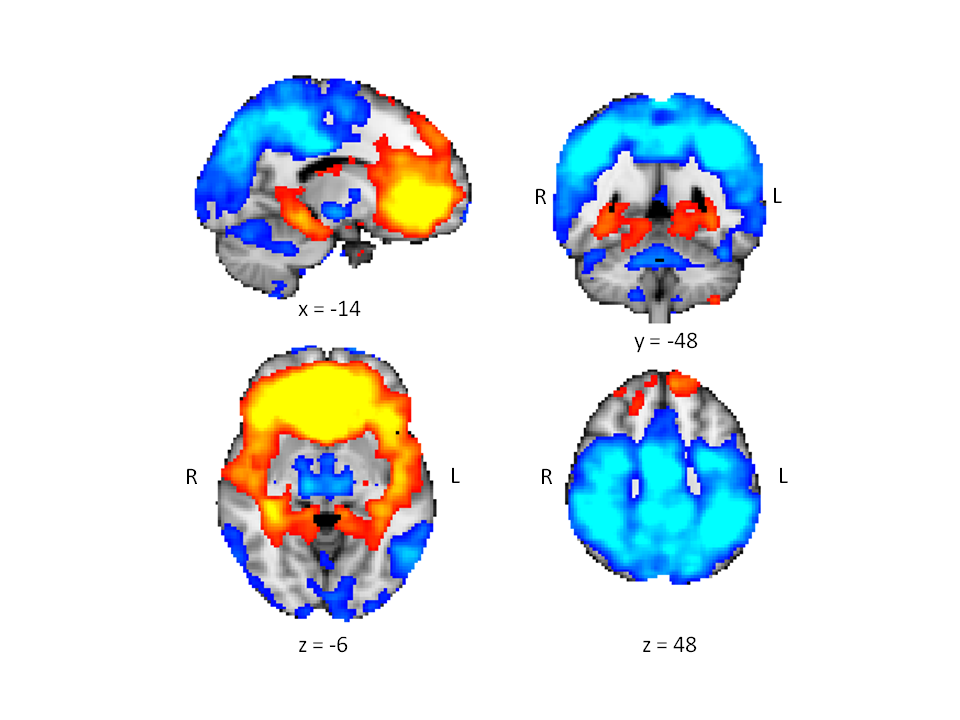


Whole-brain connectivity map of the subcallosal cingulate cortex (one sample t test of TP1, p<0.05, corrected for false discovery rate). Blue-light blue and red-yellow represent negative and positive correlations, respectively.

Figure S4.


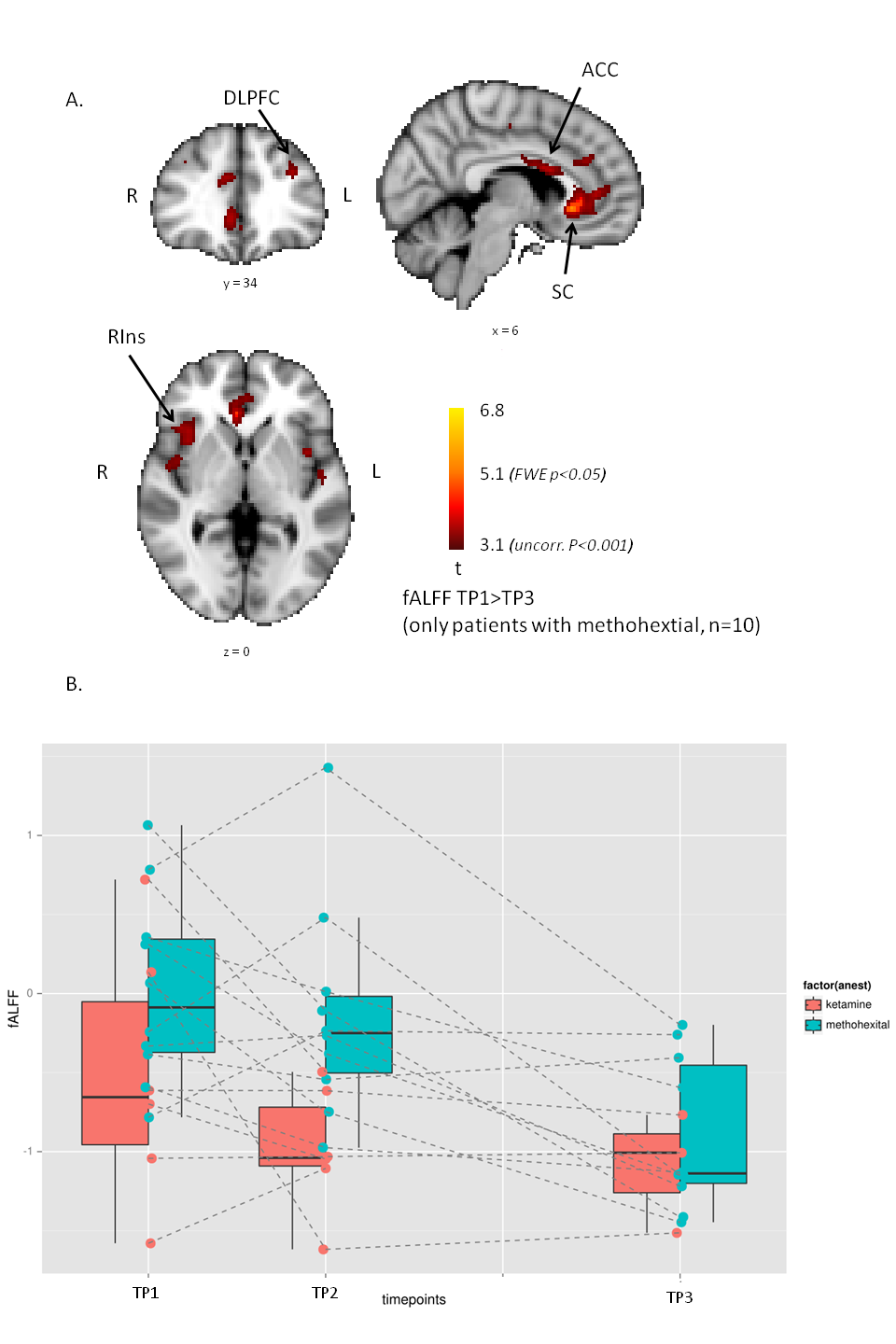


The effect of anesthetic agent. A. We reanalyzed our data using the identical approach and thresholds as shown in Figure S1, but only with patients who were on methohexital (n=10). The main findings were unchanged. B. fALFF change in SCC. Patients with ketamine and methohexital anesthesia depicted with different colors (red is ketamine, blue is methohexital). No significant differences were observed.
